# Systematic Classification of Curvature and Feature Descriptor of 3D Shape and Its Application to “Complexity” Quantification Methods

**DOI:** 10.3390/e25040624

**Published:** 2023-04-06

**Authors:** Kazuma Matsuyama, Takahiro Shimizu, Takeo Kato

**Affiliations:** 1 School of Integrated Design Engineering, Graduate School of Keio University, Yokohama 223-8522, Japan; 2 Department of Mechanical Engineering, Keio University, Yokohama 223-8522, Japan

**Keywords:** complexity, entropy, curvature, feature descriptor, generative design

## Abstract

Generative design is a system that automates part of the design process, but it cannot evaluate psychological issues related to shapes, such as “beauty” and “liking”. Designers therefore evaluate and choose the generated shapes based on their experience. Among the design features, “complexity” is considered to influence “aesthetic preference”. Although feature descriptors calculated from curvature can be used to quantify “complexity”, the selection guidelines for curvature and feature descriptors have not been adequately discussed. Therefore, this study aimed to conduct a systematic classification of curvature and a feature descriptor of 3D shapes and to apply the results to the “complexity” quantification. First, we surveyed the literature on curvature and feature descriptors and conducted a systematic classification. To quantify “complexity”, we used five curvatures (Gaussian curvature, mean curvature, Casorati curvature, shape index, and curvature index) and a feature descriptor (entropy of occurrence probability) obtained from the classification and compared them with the sensory evaluation values of “complexity”. The results showed that the determination coefficient between the quantified and sensory evaluation values of “complexity” was highest when the mean curvature was used. In addition, the Casorati curvature tended to show the highest signal-to-noise ratio (i.e., a high determination coefficient irrespective of the parameters set in the entropy calculation). These results will foster the development of generative design of 3D shapes using psychological evaluation.

## 1. Introduction

In recent years, computer-aided automatic design technology, referred to as “generative design”, has been put into practical use and applied to product design. Generative design is defined as a design system that automates part of the design process [1] or utilizes computing and manufacturing abilities to create novel and efficient designs [2]. Combined with processing technologies such as three-dimensional (3D) printers and additive manufacturing, generative design has enabled greater freedom in product design [3].

The advantage of generative design is that it enables the generation of shapes that satisfy physical characteristics such as stiffness and mass. However, its shortcoming is that psychological characteristics such as “beauty” and “preference” of the shape, which are important in the early process of a product design, are not evaluated. Therefore, designers need to evaluate and select shapes on the basis of their experience and intuition [4]. Solving this problem requires quantifying the shape features that designers focus on when evaluating a design.

When evaluating a design, designers tend to focus on macroscopic shape features, which are defined as those that appear in the entire shape as a result of combining points, lines, surfaces, and other modeling elements [5]. The main macroscopic shape features include “complexity”, “symmetry”, “order”, “proportion”, and “novelty” [6,7,8,9,10,11]. Among them, complexity and order are considered to influence “aesthetic preference” [12,13]. For example, in product design, high complexity is considered to be a factor that leads people to perceive a product as having high quality [14]. Moreover, complexity is considered a determinant of shape preference [15]. Thus, complexity is assumed to play an important role in the cognitive characteristics of shapes. Quantifying complexity will enable designers to design shapes that humans prefer.

Recent studies have indicated that a relationship exists between curvature and complexity [16,17,18]. Therefore, complexity can be quantified using a shape-feature value (hereafter referred to as a feature descriptor [19]) calculated from curvature. However, guidelines for selecting a curvature and feature descriptor to evaluate the cognition of a product shape’s complexity have not been adequately discussed. Accurately quantifying complexity requires studies on curvatures and feature descriptors of 3D shapes so that their characteristics can be understood and selection guidelines can be considered.

This study aims to perform a systematic classification of curvature and feature descriptors of 3D shapes and to apply the results to a method for quantifying complexity. This paper is organized as follows. Section 2 describes a systematic classification of curvature and feature descriptors of 3D shapes. Section 3 explains the quantification of complexity using the curvature and feature descriptor obtained from their classification. Section 4 illustrates sensory evaluation experiments that verify the applicability of the quantification. Section 5 summarizes the results and challenges of the study.

## 2. Systematic Classification of Curvature and Feature Descriptors

This section describes the systematic classification of curvature and feature descriptors of 3D shapes. We conducted a systematic classification according to a protocol known as preferred reporting items for systematic reviews and meta-analysis (PRISMA) [20]. One feature of this protocol, which is also used in the field of engineering, is that the selected references can be represented by flowcharts and tree diagrams [21,22]. The classification was conducted by three researchers from October 2021 to May 2022. The last access date to the database was 21 March 2022. 

### 2.1. Classification Method

#### 2.1.1. Keyword Setting

A paper search was conducted using the “Web of Science” database. Specifically, the search criteria were set to include all keywords related to “curvature”, “3D”, “shape”, and “shape features”, which were relevant to our study. Table 1 shows the keywords and search criteria.

#### 2.1.2. Paper Selection Based on Title and Abstract

To narrow the papers obtained in the previous subsubsection to the literature highly relevant to the present study, we carried out a paper selection process based on titles and abstracts.

1. Papers evaluating 3D shapes were extracted. That is, we excluded both papers based on two-dimensional (2D) shapes and papers based on silhouettes or images of 3D shapes.
2. Among the references extracted in procedure 1, we selected those that evaluate shapes via, for example, classification, retrieval, and recognition based on the features extracted from the shapes. Our reasoning is that these references are highly relevant to the objective of the present study, which is to evaluate the complexity of shapes. Other references involving the generation/deformation of shapes, such as by meshing, rendering, or noise detection, were eliminated.
3. Among papers extracted in procedure 2, we selected those that evaluate macroscopic features by calculating a feature descriptor for each shape, such as similarity or complexity, because these references are highly related to evaluations of the degree of complexity.

#### 2.1.3. Paper Selection Based on Content

From the papers selected as described in Section 2.1.1, we selected those based on textual content. Specifically, we selected papers that satisfy both of the following conditions:

- Curvature is calculated from a 3D shape;
- A feature descriptor is calculated on the basis of the curvature.

Papers that did not satisfy both criteria, such as those involving calculations of a curvature or feature descriptor, were excluded from our survey.

### 2.2. Classification Results

#### 2.2.1. Paper Selection

**Figure 1:**
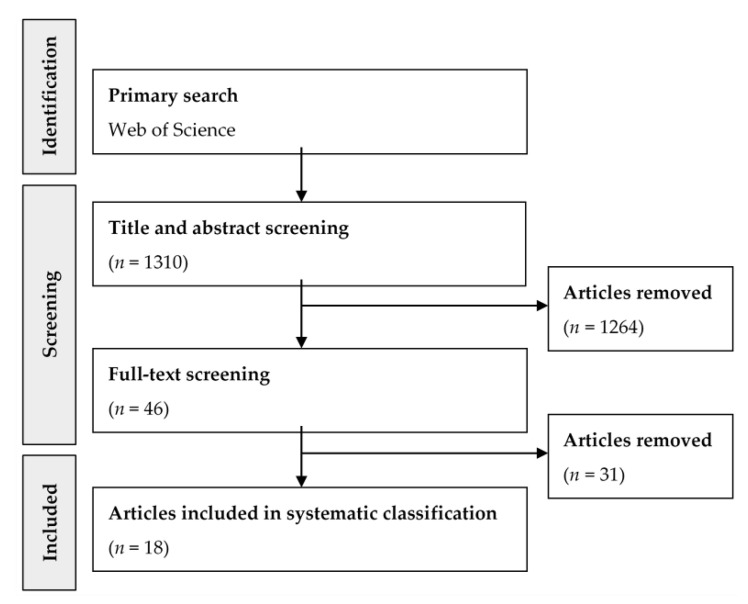
Extraction procedure.

We obtained 1310 papers as a result of keyword settings. Figure 1 shows an overview of the paper selection procedure and the number of references obtained in each procedure.

**Figure 2:**
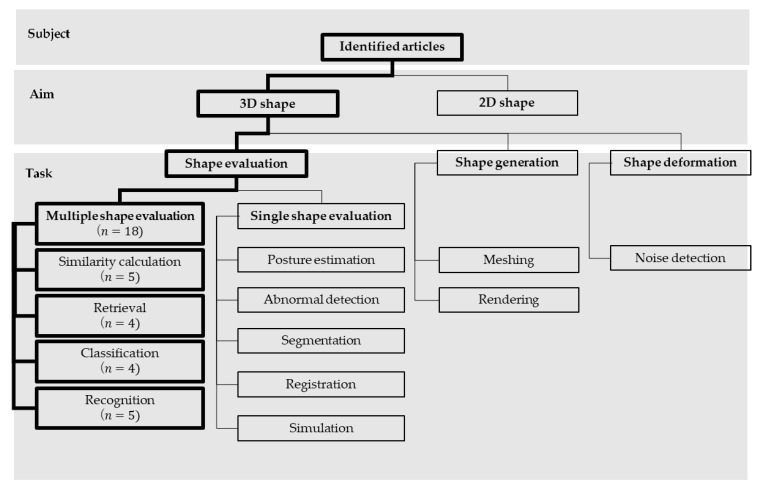
Classification of papers.

The details of the paper selection procedure are described below. First, 46 papers were selected on the basis of titles and abstracts. Figure 2 shows a tree diagram of papers classified on the basis of their titles and abstracts. The classification results are summarized as follows.

1. The shapes to be evaluated were classified into 3D and 2D shapes (generated from silhouettes or images of 3D shapes).
2. The objectives of the studies on 3D shapes were divided into three main categories: (1) shape evaluation, (2) shape generation, and (3) shape deformation. Shape evaluation is the process of extracting features from shapes and evaluating their characteristics (i.e., similarity evaluation classification, retrieval, recognition, posture estimation, registration, abnormal detection, simulation, and segmentation). Shape generation refers to the generation of 3D shapes by rendering or mesh segmentation of data such as 2D images or point clouds. Shape deformation denotes the process of removing points that deviate from the actual shape by detecting noise in the point cloud acquired by 3D scanning.
3. The tasks for shape evaluation were further classified into multiple shape evaluations (e.g., similarity calculations, classification, retrieval, and recognition) and single shape evaluations (e.g., posture estimation, registration, abnormal detection, simulation, and segmentation).

In the present study, following the criteria described in Section 2.1.2, we selected papers that targeted 3D shapes and aimed to evaluate shapes, especially by comparing them among numerous shapes. The criteria of selected papers are indicated by the bold lines in Figure 2. Finally, 46 papers were checked on the basis of the content of their text, and those without curvatures or feature descriptors were excluded, resulting in 18 papers. Table 2 lists the reference numbers, curvatures, feature descriptors, authors, and publication years of the papers.

In these papers, polygon models were used to represent 3D shapes. A polygon model is a method of representing 3D shapes using a set of minute polygons, such as triangles and quadrilaterals. Because polygon models contain information about the shape surface necessary for calculating curvature and are commonly used in the field of industrial design [23,24], polygon models are considered a useful representation method in the present study. We therefore employed polygon models.

**Table 2:** Target papers.

Reference	Curvature	Type of Feature Descriptor (Information Considered)	Author	Year
[25]	Cone curvature	Curvatures of vertices and their surroundings	Adán, M	2003
[26]	Cone curvature	Curvatures of vertices and their surroundings	Adán, M	2008
[27]	Cone curvature	Occurrence probabilities of curvatures	Adán, M	2014
[28]	Casorati curvature	Occurrence probabilities of curvatures	Wang, J	2020
[29]	Gaussian curvature Mean curvature	Clusters of curvatures	Biasotti, S	2015
[30]	Shape index Curvature index	Occurrence probabilities of curvatures	Li, B	2014
[31]	Gaussian curvature	Curvatures of vertices and their surroundings	Fu, J	2008
[32]	Shape index	Occurrence probabilities of curvatures	Jeong-Jun, S	2003
[33]	Shape index	Curvatures of vertices	Junli, Z	2014
[34]	Shape index	Curvatures of vertices and their surroundings	Li, B	2011
[35]	Curvature tensor	Curvatures of vertices	Muzahid, M	2021
[36]	Cone curvature	Transition probability	YuJie, L	2013
[37]	Mean curvature	Clusters of curvatures	Zou, K	2015
[38]	Gaussian curvature	Transition probabilities of curvatures	Guo, K	2010
[39]	Gaussian curvature	Occurrence probabilities of curvatures	Sukumar, S	2006
[40]	Gaussian curvature Mean curvature	Transition probabilities of curvatures	Guo, K	2014
[41]	Gaussian curvature	Occurrence probabilities of curvatures	Sukumar, S	2006
[42]	Gaussian curvature	Transition probabilities of curvatures	Matsumoto, T	2018

#### 2.2.2. Systematic Classification and Overview of Curvature

**Figure 3:**
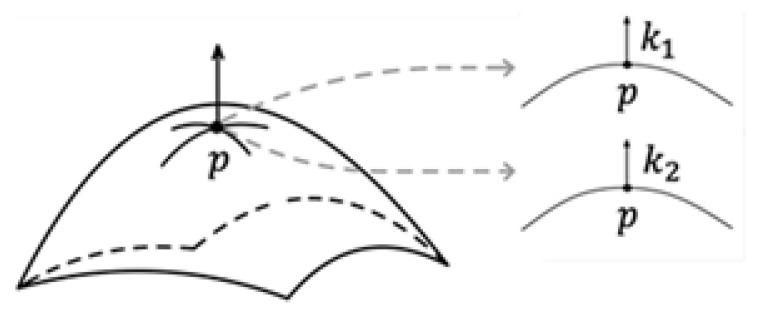
Principal curvature at point *p*.

In the 18 extracted papers, seven types of curvature (i.e., mean curvature, Gaussian curvature, shape index, curvature index, Casorati curvature, curvature tensor, and cone curvature) were used. Table 3 shows the calculation method and characteristics of each curvature. Most curvatures can be calculated using two curvatures: the maximum principal curvature $k_{1}$ and the minimum principal curvature $k_{2}$ ($k_{1}>k_{2}$) of the normal curvature created when the surface is cut at an arbitrary point (Figure 3).

#### 2.2.3. Systematic Classification and Overview of Feature Descriptor

In the 18 extracted papers, five types of feature descriptor were used. Table 4 shows the advantages and disadvantages of each feature descriptor.

- A feature descriptor that uses the curvature of each vertex of a polygonized shape is a vector whose elements are the curvatures at all vertices [33]. Compared with the other feature descriptors, a feature descriptor that uses the curvature of each vertex of a polygonized shape has the advantage of being computationally efficient but has the disadvantage of being unable to compare shapes with different numbers of vertices unless calculating statistic values (e.g., average and probability) [35].
- A feature descriptor that uses the curvature of each vertex and its surrounding vertices is a vector whose elements are calculated from the curvature of each vertex and its surrounding vertices, or it is a scalar obtained by their calculation (integration, weighted addition, etc.) [31]. Compared with other feature descriptors without the surrounding vertices, a feature descriptor that uses the curvature of each vertex and its surrounding vertices has the advantage of enabling an accurate evaluation of imperceptible surface changes but has the disadvantage of low computational efficiency [25].
- A feature descriptor that uses occurrence probabilities is a vector whose elements are the occurrence probabilities of curvature within a set range of curvatures, or entropy calculated from the vector [39]. Compared with other feature descriptors that use curvature values, a feature descriptor that uses occurrence probabilities has the advantage of allowing the user to set the range of curvatures of interest; however, it has the disadvantage that judging the validity of the curvature range setting and setting it appropriately are difficult [28].
- A feature descriptor that uses transition probability is a vector of transition probabilities (probabilities of changing from one curvature to another) between two adjacent vertices within a set curvature range, or the entropy calculated from this vector, similar to occurrence probability [39]. Compared with feature descriptors that use the occurrence probabilities, a feature descriptor that uses transition probability has the advantage of being able to accurately evaluate surfaces within a localized range because it can take into account the proportion of changes from the curvature at surrounding vertices; however, it has the disadvantage of being computationally inefficient [42].
- A feature descriptor that uses clustering methods (e.g., K-means and C-means methods) can reduce the information amount by clustering vector-type curvatures such as the cone curvature and curvature tensor to clarify the remarkable vertices of the shape. This descriptor cannot be used for scalar curvatures.

## 3. Application to a Method for Quantifying Complexity of 3D Shapes

This section describes a method for quantifying the complexity of 3D shapes. Section 3.1 describes how to calculate the five curvature indices: Gaussian curvature, mean curvature, Casorati curvature, shape index, and curvature index. Section 3.2 describes how to calculate feature descriptors using occurrence probability.

### 3.1. Methods for Calculating Curvature

**Figure 4:**
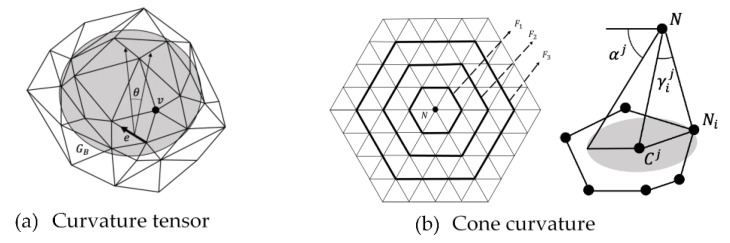
Calculation of curvatures.

This subsection describes the calculation methods of the curvatures in Table 3 from the polygon model. This study extracted five curvatures: Gaussian curvature, mean curvature, Casorati curvature, shape index, and curvature index. The curvature tensor (Figure 4a) and cone curvature (Figure 4b) were excluded for the following reasons:

- They have a large computational load to calculate the curvature. Both curvatures are expressed as an array of curvatures for a single vertex. Therefore, they are computationally more expensive than other curvatures where a single value is calculated for a single vertex. In addition, the computation required to compute feature descriptors from curvatures can also be substantial.
- They are difficult to use to evaluate curvatures of different sizes. A region (surrounding the vertex for which the curvature is calculated) must be set as a parameter. However, in actual design situations, many scenarios arise where shapes of different sizes are evaluated, and setting a common region is difficult.

**Figure 5:**
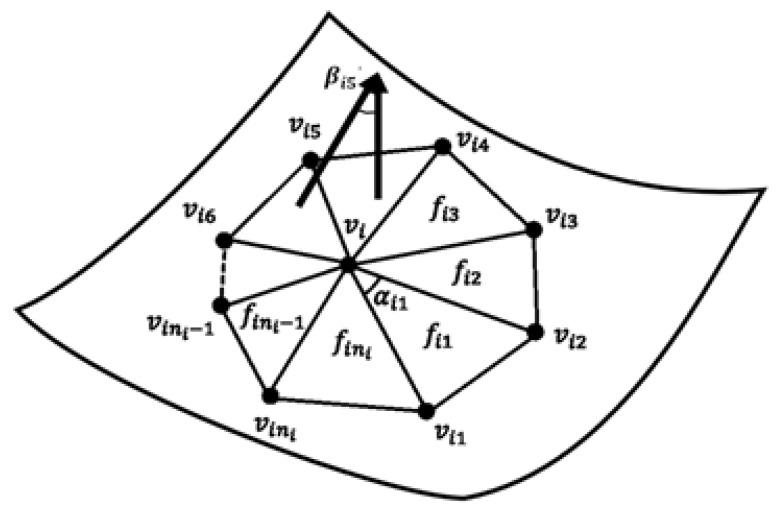
Polygons around vertex $v_{i}$.

The following illustration of the curvature calculation is in accordance with the polygon mesh in Figure 5. In this figure, $v_{i}$ is the vertex whose curvature is to be calculated, $v_{ik}\left(k=1,\;2,\ldots ,n_{i}\right)$ are vertices adjacent to vertex $v_{i}$, $f_{ik}\left(k=1,\;2,\ldots ,n_{i}\right)$ are polygons with $v_{i}$ as a vertex, and $a_{ik}$ = ∠ ($v_{ik}$, $v_{i}$, $v_{i\left(k+1\right)}$) is the angle of $f_{ik}$ at vertex $v_{i}$. 

#### 3.1.1. Gaussian Curvature

Using Gauss–Bonnet’s law and paraboloid fitting, Gaussian curvature $K_{i}$ at the *i*th vertex $v_{i}$ is calculated via the following equation [43]:

(1) $$K_{i}=\frac{2\pi -\sum_{k=1}^{n_{i}}\alpha_{ik}}{\frac{1}{3}A}$$

where A is the sum of the polygon area surrounding vertex $v_{i}$.

#### 3.1.2. Mean Curvature

Using Gauss–Bonnet’s law and paraboloid fitting, mean curvature $H_{i}$ at the *i*th vertex $v_{i}$ is calculated as follows [43]:

(2) $$H_{i}=\frac{\frac{1}{4}\sum_{k=1}^{n}\|e_{ik}\|\beta_{ik}}{\frac{1}{3}A}$$

where $e_{ik}$ is the distance between vertex $v_{i}$ and its neighboring vertices $v_{ik}\left(k=1,\;2,\ldots ,n_{i}\right)$ and $\beta_{ik}$ is the angle between adjacent polygons.

#### 3.1.3. Casorati Curvature 

The mean curvature is the curvature defined by the average of the principal curvatures, whereas the Gaussian curvature is the curvature defined by the product of the principal curvatures. Therefore, principal curvatures $k_{1}$ and $k_{2}$ can be calculated by combining Equations (1) and (2). When the maximum principal curvature is $k_{1}$ and the minimum principal curvature is $k_{2}$, both are expressed by the following equations:

(3) $$k_{1}=H+\sqrt{H^{2}-K}$$

(4) $$k_{2}=H-\sqrt{H^{2}-K}$$

The Casorati curvature ***C*** can be calculated using the principal curvatures obtained from Equations (3) and (4):

(5) $$C=\frac{\sqrt{k_{1}{}^{2}+k_{2}{}^{2}}}{2}$$

#### 3.1.4. Shape Index

The shape index can be calculated using the principal curvatures obtained from Equations (3) and (4). However, such an equation cannot be used to calculate the shape index in the plane $\left(k_{1}=k_{2}=0\right)$. Therefore, in the present study, the shape index *SI* can be calculated even for a flat surface by dividing the cases according to whether $k_{1}=k_{2}=0$ or not (Equation (6)):

(6) $$SI=\{\begin{array}{c} \frac{1}{2}-\frac{1}{\pi }arctan\left(\frac{k_{1}+k_{2}}{k_{1}-k_{2}}\right)\;\left(k_{1}\neq 0,\;k_{2}\neq 0\right)\\ \frac{1}{2}\;\left(k_{1}=k_{2}=0\right) \end{array}$$

#### 3.1.5. Curvature Index

The curvature index can be calculated by using the principal curvatures obtained in Equations (3) and (4). However, the resultant equation cannot be used to calculate the curvature index in the plane $\left(k_{1}=k_{2}=0\right)$. Therefore, in the present study, the curvature index *CI* can be calculated even for a flat surface by dividing the cases according to whether $k_{1}=k_{2}=0$ or not (Equation (7)):

(7) $$CI=\{\begin{array}{c} \frac{2}{\pi }log\left(\sqrt{\frac{k_{1}{}^{2}+k_{2}{}^{2}}{2}}\right)\;\left(k_{1}\neq 0,\;k_{2}\neq 0\right)\\ \frac{2}{\pi }log\left(\sqrt{\frac{1e-15}{2}}\right)\;\left(k_{1}=k_{2}=0\right) \end{array}$$

### 3.2. Method for Calculating Feature Descriptor

Sukumar et al. [41], who investigated systematic classification, suggested that the complexity of a shape could be quantified by considering its occurrence probability. In this approach, the Gaussian curvature at each vertex of a 3D shape is first calculated and the curvature is subsequently discretized by setting the number of states and deviation to express the continuous Gaussian curvature as discrete values. Finally, the information (occurrence probability) entropy of the discretized curvature is calculated as a feature descriptor to quantify complexity. Such discretization can be performed according to the curvature changes that can be discriminated by humans and the magnitude of the curvature that attracts their attention. Therefore, it is considered useful for evaluating human cognition. However, feature descriptors that use curvature values might be strongly affected when outliers (especially large or small curvatures) occur in a small area or when minute curvature changes that cannot be discriminated by humans occur in a wide area. Therefore, we decided to adopt a feature descriptor based on discretized occurrence probability.

Feature descriptors that consider surrounding curvature information, those that consider transition probabilities, and those that consider clustering were excluded from our calculation. Our reasoning is described below.

- The feature descriptors that consider the surrounding information are excluded because the computational load to integrate the curvature of each vertex and its neighboring vertices into a single value by calculation is larger than that of the other descriptors. In addition, the purpose of considering surrounding information is to evaluate the similarity of shapes accurately, not to evaluate human cognition.
- Feature descriptors that consider transition probabilities are excluded because evaluating the surface features of the entire shape is difficult. Although the feature descriptor can evaluate the transition of the curvature from one vertex to another, people are unlikely to focus only on local changes between vertices when evaluating complexity. In addition, checking and considering the results based on graphs such as contour plots are difficult when using transition probabilities.
- Feature descriptors that consider clustering are excluded because evaluating the curved surface features of the entire shape is difficult. The feature descriptor extracts salient points (vertices with characteristic curvature) from the entire shape as local features to evaluate the similarity among shapes deformed in only one part. However, curvatures not considered salient (not complex) are not reflected in the feature descriptors. In addition, as mentioned in the previous section, clustering can only be applied to a vector curvature (e.g., cone curvature and curvature tensors), where multiple values are calculated from a single vertex.

In this subsection, we first provide an overview of information entropy and its calculation method. We then outline the method used to calculate feature descriptors using the probability of occurrence in the present study.

#### 3.2.1. Information Entropy

Information entropy E expresses information clutter by the following equation [44]:

(8) $$E=-\overset{n}{\underset{i=1}{\sum }}p_{i}\log p_{i}$$

where n is the number of events and $p_{i}$ is the probability that event i is likely to occur. The information entropy increases as the probability of occurrence of each event is equally certain.

#### 3.2.2. Calculation Methods

1. The curvature at each vertex of the 3D shape to be evaluated is calculated as described in Section 3.1.
2. The curvature at each vertex is discretized. Specifically, the minimum area value $E_{\min}$, the maximum area value $E_{\max}$, and the number of states V are first set as parameters. The range from the minimum to the maximum area is then equally divided by the number of states to create V states $s_{i}\;\left(i=1,2,\ldots ,V\right)$. Finally, the curvature of each vertex is assigned to a state.
  (9) $$s_{1}\;(E_{\min}\;≦\;K^{\prime }\;<E_{\min}+\Delta E),\;s_{2}\;(E_{\min}+\Delta E\;≦\;K^{\prime }\;<E_{\min}+2\Delta E),\;\;\ldots ,\;s_{V}\;(E_{\max}\;-\;\Delta E\;≦\;K^{\prime }\;<\;E_{\max})\;\left(\Delta E=\frac{\left(E_{\max}-E_{\min}\right)}{V}\right)$$
3. After discretization, occurrence probability $p_{i}$ is calculated using the following equation:
  (10) $$p_{i}=\frac{N_{i}}{N}$$
  where N is the total number of vertices in the 3D shape to be evaluated and $N_{i}$ is the number of vertices assigned to state $s_{i}$.
4. The entropy of the probability of occurrence is calculated using Equation (8).

## 4. Sensory Evaluation Experiment

This section describes the sensory evaluation experiments conducted to analyze the relationship between the feature descriptors calculated from five curvatures and sensory evaluation values of complexity. We first describe the experimental method, then present the results and discuss the sensory evaluation experiments conducted on three types of shapes.

### 4.1. Experimental Methods

#### 4.1.1. Sample Shapes

In this experiment, extruded and rotated shapes, which are typical CAD shape deformation methods, were used, along with the shapes of an actual product as the “complexity” evaluation sample. The details of the shapes are described below.

- Extruded shape

**Figure 6:**
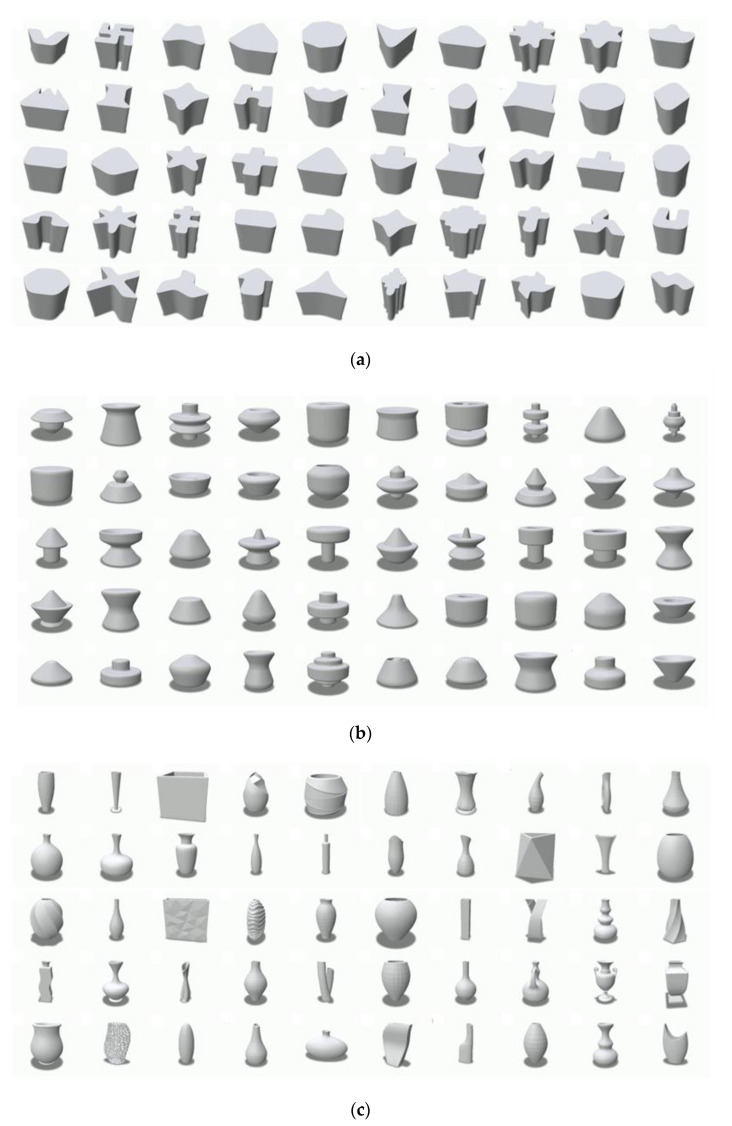
Sample shapes: (**a**) Extruded shapes; (**b**) Rotated shapes; (**c**) Vase shapes.

Extruded geometry is a 3D curved surface created from closed planar curves using extrusion, which is a 3D CAD geometry creation method. The extruded geometry was chosen for evaluation in this study because it enables the curvature characteristics of the planes, contours, and sides of an extruded surface to be compared. In this experiment, 50 2D closed curves used by Ujiie et al. [16] were edited in 3D CAD and the 3D shapes to be evaluated were created by extruding the surfaces in the vertical direction of the same shape (Figure 6a). The size of the closed planar curves was adjusted so that their maximum radius vector was equal, and the length of the extrusion was set to twice the maximum radius vector of the closed planar curves.

- Rotated shape

A rotated shape is a three-dimensional curved surface created from closed planar curves using rotation, which is a shape creation method of 3D CAD. Rotational geometry was chosen as the target of evaluation in the present study because it enables the differences in curvature among planar, convex, and concave surfaces to be considered.

The rotated shape used in this experiment was created by editing 50 closed planar curves similar to the extruded shape in 3D CAD and rotating the right side of the shape 360 degrees using the vertical direction passing through the center of gravity of the 2D shape as the rotation axis (Figure 6b). The size of the closed planar curves was adjusted so that the maximum radius vectors of the curves were equal.

- Vase shape

A vase is a product with a high degree of freedom in its shape, and various types of curved surfaces can appear. Therefore, using the vase shape as the evaluation target, we can consider the influence of various curved surfaces on the perception of complexity. In addition to its functional role of holding cut flowers, a vase also plays a role as an interior decoration; thus, it is important to take aesthetic preferences into account when modeling [45]. A vase is therefore considered an appropriate target for evaluation in the present study, which aims at aesthetic preferences in product shape.

A total of 50 vase shapes were used in this experiment: 25 vase shapes provided by Free3D [46] and 25 vase shapes provided by CGTrader [47]; the top of each vase was closed with a flat surface to prevent curvature from occurring inside the vase, which is not visible (Figure 6c).

#### 4.1.2. Experimental Conditions

- Evaluation method: A 5-point Likert scale was used for complexity: “low complexity: 1”, “somewhat low complexity: 2”, “undecided: 3”, “somewhat high complexity: 4”, and “high complexity: 5”.
- Presentation method: An online questionnaire was used, with the sample shape colored gray and displayed on a white background. In addition, 10 of each sample shape were randomly selected and presented in duplicate to check the accuracy of the participants’ evaluation. Each shape was rotated at a constant speed (*z*-axis: 9.0 s per rotation).
- Participants: 110 male and 110 female participants. To exclude participants with inaccurate evaluations, we calculated the absolute value of the difference in sensory evaluation values among the 10 shapes presented in duplicate and excluded participants whose sum was 11 or more. As a result, data from 81 participants for the extruded shape, 89 participants for the rotated shape, and 61 participants for the vase shape were used.

To calculate the curvature and feature descriptors from the geometry under evaluation, we performed the following additional procedures in the present study.

1. Equal division of polygons

The shapes created by the method described in Section 4.1.1 had polygons with nonuniform shapes and sizes. Because participants were expected to evaluate complexity from the entire shape, each polygon vertex should be uniformly distributed. In addition, because the area of polygons was involved in the formulas for calculating Gaussian curvature and mean curvature, the curvature value might be affected by the size of the area.

Therefore, in the present study, polygons were equally divided by applying the advancing-front method to the created geometry. An advantage of the advancing-front method is that the polygons are closer to equilateral triangles than those used in other methods of polygon division, such as the Delaunay division and bubble mesh [48]. In the present study, equal division was performed using software [49] that implements the method, and the results were analyzed for 50 extruded shapes, 46 rotated shapes, and 38 vase shapes for which equal division was possible.

2. Determination of discretization parameters

When calculating the entropy of occurrence probability, discretization is required to divide the continuous curvature into multiple states. In the present study, suitable ranges were set for each shape and curvature by the following methods:

1. The curvature was calculated at all vertices of the shape to be evaluated.
2. A percentile range [$E_{0.003\%}$, $E_{99.997\%}$] that included curvatures between 0.003% and 99.997% of the calculated curvatures was calculated. This range was determined with reference to the range of (mean) ± 4 (standard deviation) for the normal distribution.
3. The curvatures included in the determined percentile range [$E_{0.003\%}$, $E_{99.997\%}$] were divided by V, as in the conventional method [39]. The curvatures above the maximum and below the minimum in the same range were assigned to the state with the largest and smallest curvatures, respectively, as in the method of Matsumoto et al. [42].
4. Integers from 2 to 20 were used as candidates for the number of states.

### 4.2. Experimental Results

#### 4.2.1. Extruded Shape

**Figure 7:**
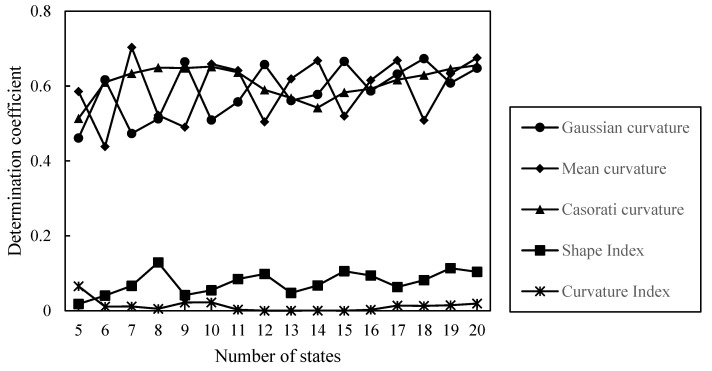
Relationship between the number of states and determination coefficient in extruded shapes.

Table 5 shows the results obtained when calculating the entropy of occurrence probability using five different curvatures for extruded shapes. The table lists as discretization parameters the maximum range $E_{\max}$, the minimum range $E_{\min}$, and the number of states V when the determination coefficient of the logarithmic approximation between the sensory evaluation values and the entropy is maximal. Note that the logarithmic approximation is applied based on Fechner’s law, which indicates the relationship between human sensitivity and stimuli using the logarithmic function and used in shape cognition studies [17,18,42,50]. In addition, to evaluate the stability of the discretization, the table lists the average, standard deviation, and the larger-the-better signal-to-noise (SN) ratio of the determination coefficients for the range of 5 to 20 states. Figure 7 shows the relationship of the determination coefficients with complexity versus the number of states. Table 5 and Figure 7 indicate that the highest correlation is obtained when the mean curvature is used, with a determination coefficient of 0.704. However, the determination coefficient is stable with respect to the number of states (the SN ratio is highest) when the Casorati curvature is used.

#### 4.2.2. Rotated Shape

**Figure 8:**
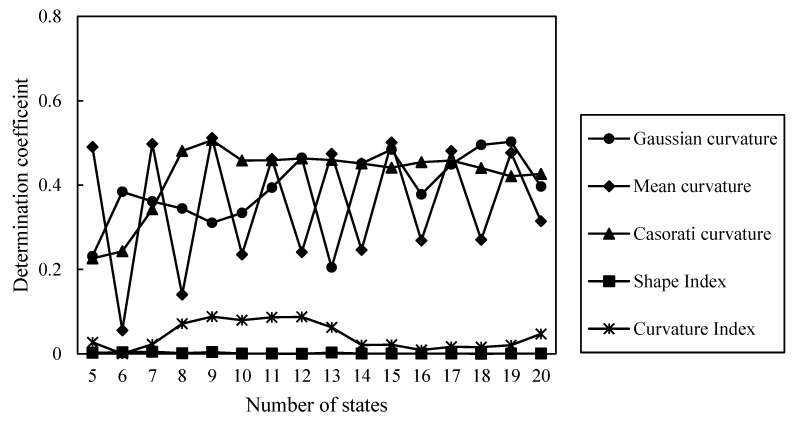
Relationship between the number of states and determination coefficient in rotated shapes.

Table 6 shows the results of calculations of the entropy of occurrence probability using five different curvatures for rotated shapes. Figure 8 shows the determination coefficient between complexity and the number of states. Table 6 and Figure 8 indicate that the highest correlation is obtained when the mean curvature is used, with a determination coefficient of 0.551. However, the determination coefficient is stable with respect to the number of states (the SN ratio is highest) when the Casorati curvature is used.

#### 4.2.3. Vase Shape

**Figure 9:**
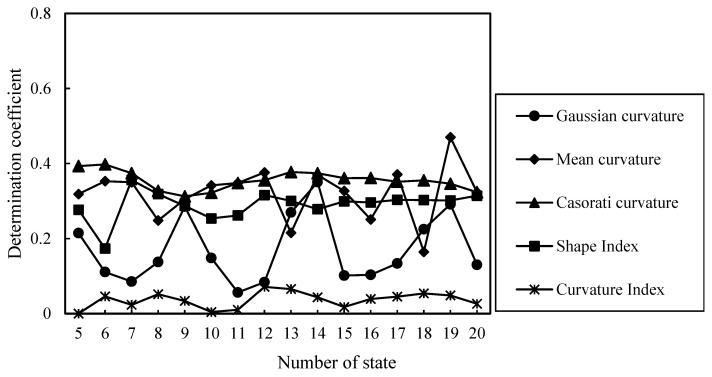
Relationship between the number of states and determination coefficient in extruded shapes.

Table 7 shows the results of calculations of the entropy of occurrence probability using five types of curvature for vase shapes. Figure 9 shows the determination coefficient between the number of states and complexity. Table 7 and Figure 9 indicate that the highest correlation is obtained when the mean curvature is used, with a determination coefficient of 0.471. However, the determination coefficient is stable with respect to the number of states (the SN ratio is highest) when the Casorati curvature is used.

### 4.3. Experimental Discussion

#### 4.3.1. Variation in Determination Coefficients with Different Numbers of States

When the Gaussian curvature and mean curvature were used, the determination coefficient with complexity tended to vary with the number of states V. This variation was observed in all three sample shapes, suggesting that the characteristics of the two curvatures affect the result. In the following, we focus on the number of states with large and small determination coefficients for the Gaussian curvature and mean curvature and discuss the reasons for the variation.

1. Gaussian curvature

**Figure 10:**
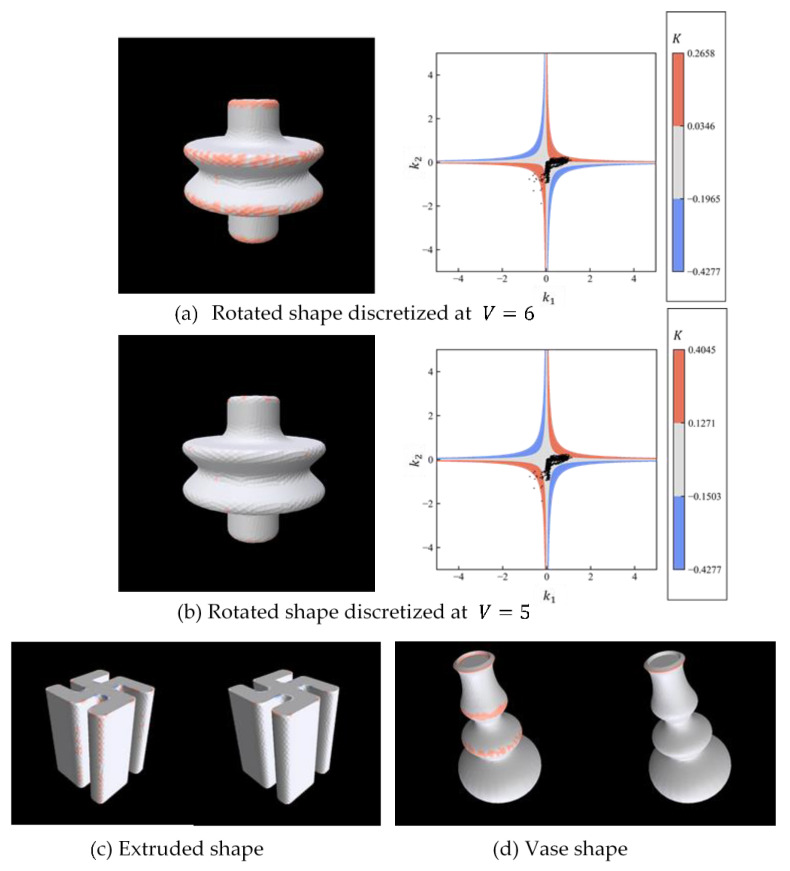
Shapes with large entropy changes between the number of states: (**a**) contour diagram of the Gaussian curvature with scatter plots of the principal curvatures of a rotated shape (*V* = 6); (**b**) ditto (*V* = 5); (**c**) an extruded shape example whose entropy variation is large between the number of states; (**d**) a vase shape example whose entropy variation is large between the number of states.

As an example, we compare the number of states 6 with 5, where the change in the determination coefficient is large in rotated shapes. In the rotated shape, where the entropy variation is particularly large, the surface is discretized for each number of states as follows (Figure 10):

- For V=6 with a high determination coefficient, flat surfaces and surfaces with a principal curvature of 0 at one side are discretized into gray states, whereas convex surfaces are discretized into red states (Figure 10a).
- For V=5 with a low determination coefficient, most of the surfaces are in the gray states (Figure 10b).

In rotational shapes, steep convex surfaces tend to influence the evaluation of complexity. Therefore, the number of states 6 that can discriminate complex surfaces (convex surface) from uncomplex surfaces (i.e., the surfaces whose maximum and/or minimum principal curvature is almost 0) is consistent with the human perception of complexity and is considered to have a high determination coefficient. Similarly, in the extruded and vase shapes, the number of states that discriminate complex and uncomplex surfaces tends to have a high determination coefficient (Figure 10c,d).

2. Mean curvature

**Figure 11:**
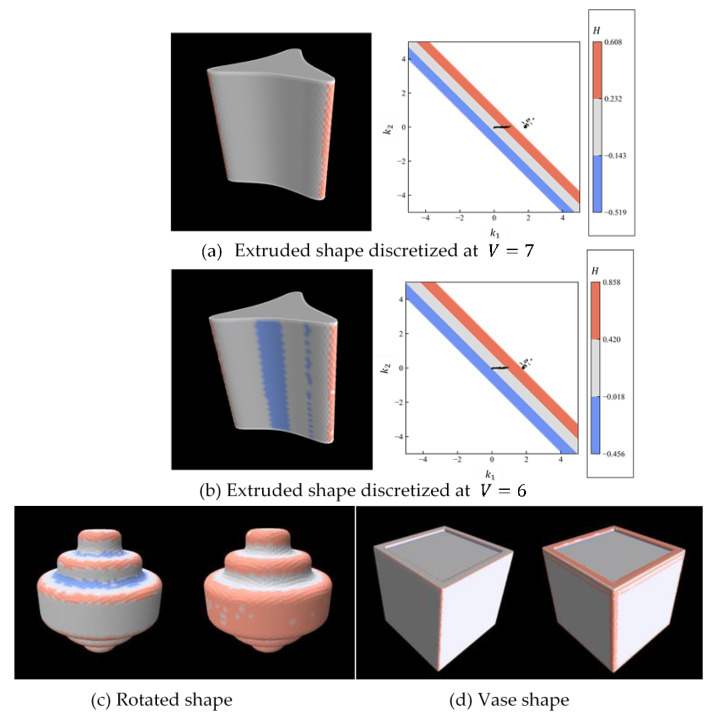
Shape with large entropy changes between the number of states: (**a**) contour diagram of the mean curvature with scatter plots of the principal curvatures of an extruded shape (*V* = 7); (**b**) ditto (*V* = 6); (**c**) a rotated shape example whose entropy variation is large between the number of states; (**d**) a vase shape example whose entropy variation is large between the number of states.

As an example, we compare the number of states 7 with 6, where the change in the determination coefficient is large in the extruded shapes. In the extruded shapes, where the entropy variation is particularly large, the surface is discretized for each number of states as follows (Figure 11):

- For V=7 with a high determination coefficient, flat surfaces and moderate surfaces with a principal curvature of 0 at one side are discretized into gray states. However, steep surfaces with a principal curvature of 0 at one side are discretized into red areas (Figure 11a).
- For V=7 with a high determination coefficient, flat surfaces and steep surfaces with a principal curvature of 0 at one side are discretized into gray states. However, moderate surfaces with a principal curvature of 0 at one side are discretized into blue areas (Figure 11b).

#### 4.3.2. Variation in Determination Coefficients with Different Curvatures

The experimental results show that the Casorati curvatures are stable and highly correlated for all the investigated shapes. However, depending on the number of states, the determination coefficients of the Gaussian curvature and mean curvature can exceed the determination coefficient of the Casorati curvature. Therefore, we discuss the factors by comparing the determination coefficients of the Gaussian curvature and mean curvature with those of the Casorati curvature at the number of states where the determination coefficients of the Gaussian curvature and mean curvature are maximal.

1. Gaussian curvature

**Figure 12:**
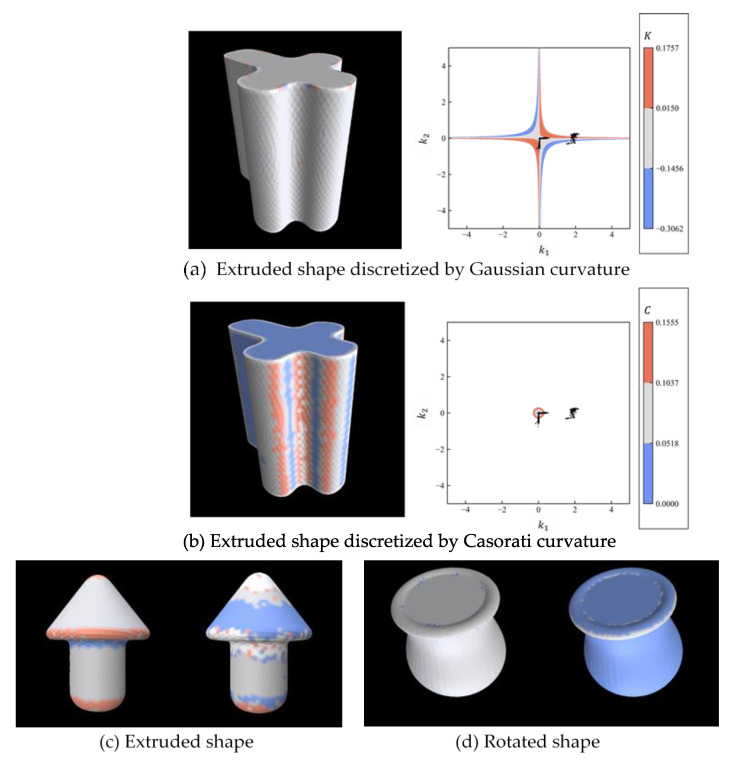
Shape with large entropy changes between curvatures: (**a**) contour diagram of the Gaussian curvature with scatter plots of the principal curvatures of an extruded shape; (**b**) contour diagram of the Casorati curvature with scatter plots of the principal curvatures of an extruded shape; (**c**) an extruded shape example whose curvature variation is large between the Gaussian and Casorati curvatures; (**d**) a vase shape example whose curvature variation is large between the Gaussian and Casorati curvature.

As an example, in the extruded geometry, the highest determination coefficient when using the Gaussian curvature is found for a number of states 18. We focus on the extruded shape, where the entropy change between the two curvatures is large. At each curvature, the surfaces of extruded shape are discretized as follows (Figure 12).

- For the Gaussian curvature, moderate surfaces with a principal curvature of 0 at one side and flat surfaces are discretized into gray states.
- For the Casorati curvature, moderate surfaces with a principal curvature of 0 at one side are discretized into different states: blue, gray, and red.

Unlike the Gaussian curvature, the Casorati curvature can take positive values in the column plane. In addition, compared with the Gaussian curvature, the Casorati curvature tends to be larger for small (near zero) changes in principal curvature. Therefore, for extruded shapes, the Gaussian curvature is expected to quantify complexity more accurately than the Casorati curvature.

2. Mean curvature

**Figure 13:**
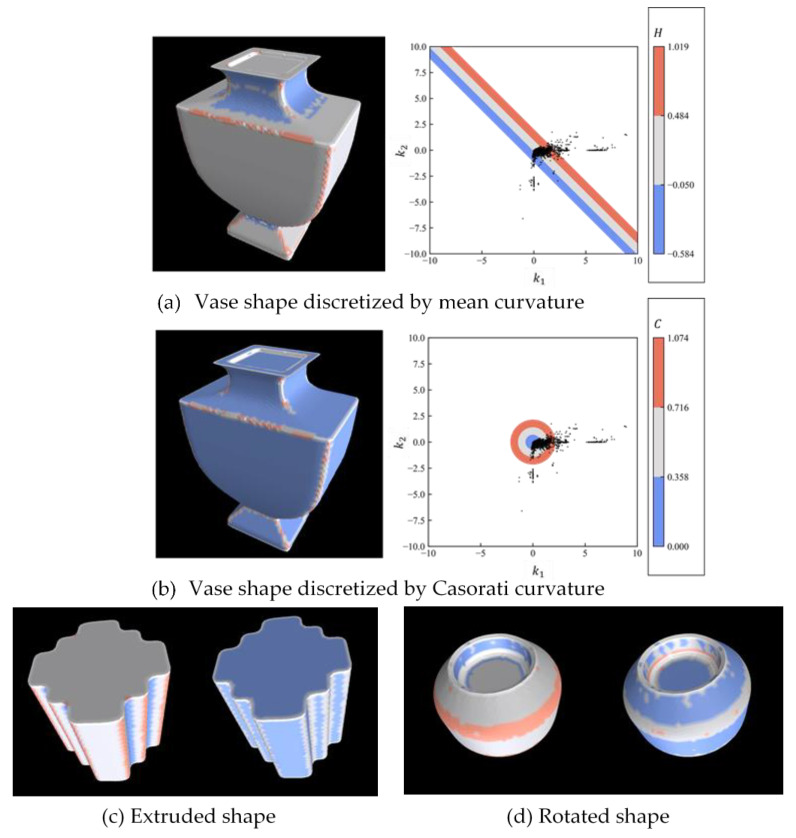
Shape with large entropy changes between curvatures: (**a**) contour diagram of the mean curvature with scatter plots of the principal curvatures of a vase shape; (**b**) contour diagram of the Casorati curvature with scatter plots of the principal curvatures of an extruded shape; (**c**) an extruded shape example whose curvature variation is large between the Gaussian and Casorati curvatures; (**d**) a rotated shape example whose curvature variation is large between the Gaussian and Casorati curvature.

As an example, for the vase shape, the highest determination coefficient when using the mean curvature is found when the number of states is 19. We focus on the vase shape, where the entropy change between the two curvatures is large. For each curvature, the surfaces of the vase shape are discretized as follows (Figure 13):

- For the mean curvature, flat and principal curvatures of 0 at one side are discretized into gray states, whereas convex edges are assigned to the red area and concave edges to the blue area.
- For the Casorati curvature, flat surfaces and concave surfaces are discretized into the same blue area. Convex surfaces as edges are assigned to gray or red states.

Vase shapes tend to have a higher complexity evaluation when both convex and concave surfaces are included. Therefore, mean curvatures with different states for convex and concave surfaces are considered appropriate.

#### 4.3.3. Variation in Determination Coefficients with Different Feature Descriptors

In the present study, the entropy of occurrence probability was calculated as a feature descriptor because it can be used to evaluate the diversity of curvature and because the discretization parameters can be set according to human cognition. To verify the validity of this feature descriptor, we calculated the curvature moment as a comparison target.

The curvature moment is a characteristic value calculated by considering the curvature group calculated from the shape as a probability distribution. Because some studies have evaluated the similarity of shapes using moments of curvature [51], it is applicable to the quantification of complexity in the present study. Here, the average, standard deviation, coefficient of variation, kurtosis, and skewness were calculated as moments.

Table 8 shows the determination coefficients when the entropy of occurrence probability is used and when the curvature moments are used. The table shows the average, standard deviation, and desired characteristic SN ratio of the 15 determination coefficients (3 shapes × 5 curvatures) calculated for each feature descriptor. The results in this table show that the occurrence probability has large and stable determination coefficients with complexity.

**Figure 14:**
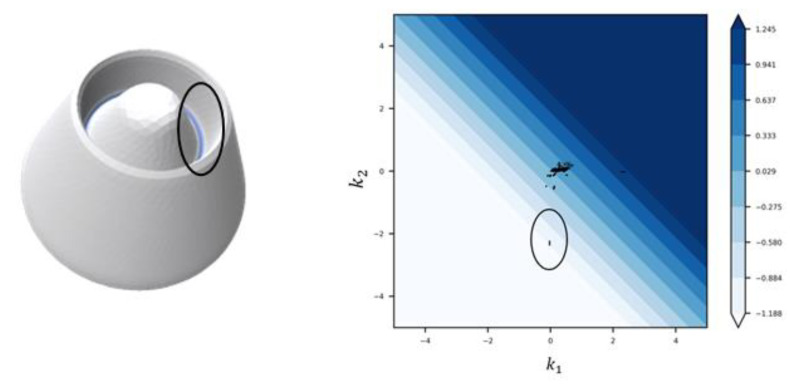
Shape with large difference between the sensory evaluation value and the curvature moment (standard deviation).

The lower determination coefficient when using the curvature moment is attributed to the effect of mesh irregularities. In this subsection, we focus on the standard deviation that exhibits the highest desirability characteristic SN ratio among the curvature moments. The shape shown in Figure 14 is evaluated to be of medium complexity; however, the standard deviation is large. This shape is uneven in the blue area in Figure 14, and the standard deviation is considered to have been increased because of the effect of this area. Because the occurrence probability entropy is discretized, the curvature value does not affect the entropy value. Therefore, we speculate that the high determination coefficient is attributable to the fact that the effect of the curvature of such outliers can be reduced.

One limitation of this study is that only the determination coefficient of the logarithmic approximation between the quantified and sensory evaluation values of complexity was used to evaluate their correlation. Although we confirmed some scatter diagrams (Appendix A) and this procedure is assumed to be valid, other procedures can be more suitable. 

## 5. Conclusions

We conducted a systematic classification of curvature and feature descriptors in 3D shapes. The results showed that Gaussian curvature, mean curvature, Casorati curvature, curvature index, shape index, curvature tensor, and cone curvature can be used as curvatures calculated for 3D shapes. In addition, we confirmed that “feature descriptors considering surrounding information”, “feature descriptors considering occurrence probability”, “feature descriptors considering transition probability”, and “feature descriptors considering clustering” could be used as feature descriptors calculated for the 3D shapes.

We applied the results of the systematic classification to a method for quantifying the “complexity” of 3D shapes. Specifically, Gaussian curvature, mean curvature, Casorati curvature, curvature index, and shape index were calculated for the polygon model. In addition, the probability of occurrence (information) entropy of these five types of curvatures was calculated as a feature descriptor.

Finally, we conducted sensory evaluation experiments to verify the validity of the quantification method; the results showed that the highest determination coefficient for all three shapes was obtained when the mean curvature was used. To investigate the effect of discretization when calculating entropy, we calculated the mean value, standard deviation, and the SN ratio of the determination coefficient when the number of states was varied. The results showed that the Casorati curvature was stable for almost all of the investigated shapes.

Our future work will include (1) conducting sensory evaluation experiments using other sample shapes to verify whether the same trend can be confirmed and (2) constructing a comparative criterion to enable a comparison that includes other types of curvatures and feature descriptors excluded in the present study.

## Figures and Tables

**Table 1:** Keywords and search criteria.

Curvature	AND/OR	3D	AND/OR	Shape	AND/OR	Feature
“Curvature”	AND	“3d”	AND	“Shape”	AND	“Preference”
		OR		OR		OR
		“Three-dimensions”		“Form”		“Aesthetic”
				OR		OR
				“Appearance”		“Likeness”
				OR		OR
				“Outline”		“Liking”
				OR		OR
				“Silhouette”		“Complex”
						OR
						“Complexity”
						OR
						“Novelty”
						OR
						“Similarity”
						OR
						“Order”

**Table 3:** Classification of curvatures.

Curvature	Calculation Method	Properties	References
Mean curvature	$H=\frac{k_{1}+k_{2}}{2}$	Identify convex and concave surfaces	[29,37,40]
Gaussian curvature	$K=k_{1}k_{2}$	Identify saddle surface as negative values Calculate 0 even on cylindrical surfaces	[29,31,38,39,40,41,42]
Casorati curvature	$C=\frac{\sqrt{k_{1}{}^{2}+k_{2}{}^{2}}}{2}$	Identify saddle and uneven surfaces Cannot be calculated on a flat surface	[28]
Shape index	$SI=\frac{1}{2}-\frac{1}{\pi }\arctan\left(\frac{k_{1}+k_{2}}{k_{1}-k_{2}}\right)$	Expressed in absolute value Cannot be calculated on a flat surface	[30,32,33,34]
Curvature index	$CI=\frac{2}{\pi }\log\left(\sqrt{\frac{k_{1}{}^{2}+k_{2}{}^{2}}{2}}\right)$	Identify flat and curved surface Expressed in absolute value	[30]
Curvature tensor	$\tau_{v}=\frac{1}{G_{A}}\underset{N_{e}}{\sum }\theta \cdot L_{G_{B}}\cdot \bar{e}\cdot {\bar{e}}^{t}$	Identify flat and curved surface Appropriate area setting is necessary	[35]
Cone curvature	$\alpha_{j}=\mathrm{sign}\left(F^{j}\right)\left|\frac{\pi }{2}-\frac{1}{t_{j}}\overset{t_{j}}{\underset{i=1}{\sum }}r_{i}^{j}\right|$	Identify flat and curved surface Smoothly calculated	[25,26,27,36]

$G_{B}$: circular region around a vertex; $G_{A\;}$: area of $G_{B}$; $N_{e}$: number of edges; θ: angle between two triangles; $L_{G_{B}}$: length of the part of the edge inside $G_{B}$; e: edge of triangles; $\bar{e}$: unit vector of e; ${\bar{e}}^{t}$: transpose vector of e; $F^{j}$: *j*th modeling wave $\mathrm{sign}\left(F^{j}\right)$: sign function depends on convex–concave at $F^{j}$; $t_{j}$: number of vertices at $F^{j}$; $r_{i}^{j}$: angle of $∠N_{i}NC^{j}$.

**Table 4:** Classification of curvatures.

Information Considered	Advantages/Disadvantages	References
Curvatures of vertices	Feature descriptors can be calculated from curvatures with a small amount of computation. Comparisons between shapes require matching the number of vertices and the order in which curvatures are stored in the array.	[33,35]
Curvatures of vertices and their surroundings	Using the curvature of a vertex and its surrounding vertices, this feature descriptor takes the surface features of adjacent vertices into account. This consideration of surface features increases the computational complexity because the curvature must be calculated for multiple vertices.	[24,25,26,31,34,36]
Occurrence probabilities of curvatures	Using the distribution of curvatures that appear, the diversity of curvatures is considered. The curvature must be discretized by setting appropriate parameters in advance.	[27,28,30,32,34,37,38,39,40,41]
Transition probabilities of curvatures	Using the curvature of two vertices that are adjacent or arbitrarily far apart, surface features between the vertices are considered. The curvature must be discretized by setting appropriate parameters in advance.	[42]
Clusters of curvatures	By classifying curvatures based on the similarity of values and using only some groups of curvatures as feature descriptors, shape features are expressed in a short array. Because only some vertices and curvatures are used, detailed features of the surface might not be represented.	[29,30,34,36,37]

**Table 5:** Experimental results for extruded shapes.

Curvature	Parameter			Determination Coefficient			
	*V*	*E_max_*	*E_min_*	Maximum	Average	Standard Deviation	SN Ratio
Gaussian curvature	18	1.943	−0.949	0.666	0.588	0.068	−0.804
Mean curvature	7	1.734	−0.894	0.704	0.591	0.079	−4.826
Casorati curvature	10	1.477	0.000	0.652	0.610	0.041	−4.350
Shape index	8	1.000	0.000	0.0.129	0.076	0.030	−26.167
Curvature index	5	0.737	−11.785	0.022	0.013	0.016	−90.085

**Table 6:** Experimental results for rotated shapes.

Curvature	Parameter			Determination Coefficient			
	*V*	*E_max_*	*E_min_*	Maximum	Average	Standard Deviation	SN Ratio
Gaussian curvature	19	0.959	−0.428	0.503	0.387	0.086	−9.144
Mean curvature	7	1.245	−1.188	0.551	0.354	0.144	−14.918
Casorati curvature	9	1.200	0.000	0.501	0.421	0.078	−8.297
Shape index	7	1.000	0.000	0.005	0.001	0.002	−82.639
Curvature index	9	0.340	−11.215	0.088	0.042	0.030	−61.944

**Table 7:** Experimental results for vase shapes.

Curvature	Parameter			Determination Coefficient			
	*V*	*E_max_*	*E_min_*	Maximum	Average	Standard Deviation	SN Ratio
Gaussian curvature	14	17.652	−4.935	0.351	0.170	0.087	−18.629
Mean curvature	19	6.364	−3.791	0.471	0.321	0.070	−10.731
Casorati curvature	6	6.799	0.000	0.398	0.355	0.024	−9.054
Shape index	7	1.000	0.000	0.356	0.290	0.038	−11.093
Curvature index	13	1.441	−11.214	0.066	0.036	0.020	−67.962

**Table 8:** Comparison between entropy of occurrence probability and curvature moment.

Feature Descriptor		Average	Standard Deviation	SN Ratio
Moment	Mean	0.142	0.307	−44.786
	Standard deviation	0.307	0.247	−38.357
	Coefficient of variation	0.210	0.183	−43.974
	Kurtosis	0.165	0.241	−50.722
	Skewness	0.153	0.211	−58.771
Entropy of occurrence probability		0.393	0.222	−34.310

**Figure A1:**
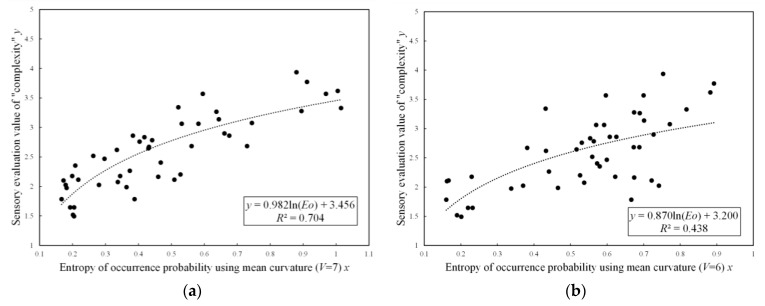
Scatter diagrams with entropy values using mean curvature on the x axis and the sensory evaluation values on the y axis: (**a**) the number of states *V* = 7; (**b**) *V* = 6.

## Data Availability

Not applicable.

## Appendix A

Figure A1 shows the relationship between the entropy using mean curvature and sensory evaluation values.
